# Stable Jumping Control Based on Deep Reinforcement Learning for a Locust-Inspired Robot

**DOI:** 10.3390/biomimetics9090548

**Published:** 2024-09-11

**Authors:** Qijie Zhou, Gangyang Li, Rui Tang, Yi Xu, Hao Wen, Qing Shi

**Affiliations:** 1 Intelligent Robotics Institute, School of Mechatronical Engineering, Beijing Institute of Technology, Beijing 100081, China; zhou_qijie@bit.edu.cn (Q.Z.); 3120230140@bit.edu.cn (G.L.); 3120225144@bit.edu.cn (R.T.); xuy@bit.edu.cn (Y.X.); 2 Key Laboratory of Biomimetic Robots and Systems, Beijing Institute of Technology, Ministry of Education, Beijing 100081, China; 3 School of Mathematics and Computational Science, Xiangtan University, Xiangtan 411105, China; wenh88467@gmail.com; 4 Yangtze Delta Region Academy, Beijing Institute of Technology, Jiaxing 314000, China

**Keywords:** biologically inspired robots, dynamic stability, deep reinforcement learning

## Abstract

Biologically inspired jumping robots exhibit exceptional movement capabilities and can quickly overcome obstacles. However, the stability and accuracy of jumping movements are significantly compromised by rapid changes in posture. Here, we propose a stable jumping control algorithm for a locust-inspired jumping robot based on deep reinforcement learning. The algorithm utilizes a training framework comprising two neural network modules (actor network and critic network) to enhance training performance. The framework can control jumping by directly mapping the robot’s observations (robot position and velocity, obstacle position, target position, etc.) to its joint torques. The control policy increases randomness and exploration by introducing an entropy term to the policy function. Moreover, we designed a stage incentive mechanism to adjust the reward function dynamically, thereby improving the robot’s jumping stability and accuracy. We established a locus-inspired jumping robot platform and conducted a series of jumping experiments in simulation. The results indicate that the robot could perform smooth and non-flip jumps, with the error of the distance from the target remaining below 3%. The robot consumed 44.6% less energy to travel the same distance by jumping compared with walking. Additionally, the proposed algorithm exhibited a faster convergence rate and improved convergence effects compared with other classical algorithms.

## 1. Introduction

Biologically inspired robots offer the potential to assist or replace humans in tedious and dangerous tasks, demonstrating increased efficiency, accuracy, and repeatability by virtue of their sophisticated structural designs and flexible movement patterns [1]. These robots have significant application prospects in search and rescue, environmental exploration, and smart cities [2,3]. Among them, bionic jumping robots have garnered considerable attention because of their superior environmental adaptability, which is facilitated by their unique jumping motion. Jumping, as a significant type of locomotion, presents several advantages over traditional walking and crawling methods, including high energy density and increased efficiency in traversing obstacles [4]. The diverse range of jumping mechanisms observed in various insects and animals provides valuable biological templates for the design and optimization of bionic jumping robots. By studying these natural models, researchers can enhance the performance and versatility of robotic systems, paving the way for advancements in robotics that more effectively mimic the agility and adaptability found in nature [5].

Common bionic jumping robots include locust [6], flea [7], and galago robots [8]. Mo et al. developed a locust-inspired jumping robot by analyzing kinematic and dynamic stability factors to optimize jumping performance [9]. Jung et al. proposed a steerable jumping robot that mimics a hopping frog’s power-producing hind legs and moment cancelation to achieve directional control [10]. By imitating the physiological structure and jumping mechanism of wax cicadas, Bai et al. designed a parallel single-degree-of-freedom double six-link jumping robot [11]. Zhakypov et al. created a swarm of multimodal millirobots called Tribot, capable of crossing barriers and performing cooperative transportation [12]. Yang et al. presented a variable energy storage and release mechanism, inspired by locusts’ hind legs, along with a cross structure for flexible jump trajectory control and take-off attitude stabilization [13]. However, these jumping robots have bionic configurations that do not accurately control jump speed or angle, resulting in frequent flipping during the dynamic jump process.

Controlling bionic robots to perform agile locomotion remains a substantial challenge in the field of robotics [14]. Model predictive control [15] and trajectory optimization [16,17] have offered productive approaches to establish the kinematic and dynamic models of robots manually, but the model construction process is time-consuming and laborious. On the other hand, deep reinforcement learning (DRL) offers a promising approach for robots to learn highly agile dynamic motions. DRL shifts the focus from constructing precise kinematic and dynamic models of robots to enabling them to learn policies for motion control through interaction with their environment [18]. Deep Q-Learning Networks (DQNs) [19] can handle high-dimensional state inputs by using deep neural networks, which is difficult to achieve with traditional Q-learning. However, applying DQNs to the continuous action space of robot motion control poses challenges. The actor–critic architecture is particularly well suited for addressing the challenges associated with continuous action spaces; it consists of two primary components: the actor network, which is responsible for selecting actions and updating policies, and the critic network, which assesses the value of actions and provides feedback to improve the performance of the actor network. The actor–critic architecture enables more effective learning of complex tasks by combining policy optimization with value evaluation. Several classical DRL algorithms based on the actor–critic architecture have been proposed, including Deep Deterministic Policy Gradient (DDPG) [20], Advantage Actor–Critic (A2C) [21], and Proximal Policy Optimization (PPO) [22]. DDPG is a policy gradient-based method that can be implemented in high-dimensional continuous action spaces but struggles with low training stability and exploration efficiency. A2C improves learning stability by combining policy gradients with value functions, but it comes with higher computational complexity and depends heavily on the accuracy of the critic’s estimates. PPO increases policy stability by introducing limiting probability ratios; however, in high-dimensional and complex tasks, it may exhibit slow convergence. Several researchers have applied these DRL algorithms in conjunction with biologically inspired actions to address complex motion control problems in robotics. Smith et al. incorporated data from existing controllers as transitions in the replay buffer for a training policy [23]. Inspired by the human learning process, Ye et al. applied a reference signal directly as a feedforward action, which was combined with a feedback action learned based on DRL to generate the final action policy [24]. DRL can be used to instruct a multi-joint robot to learn the behavior of animals by constructing reward functions that are based on biological data collected in nature. Liu et al. trained humanoid robots to imitate pretrained football-specific skills by using human motion capture data [25]. All of the above work involves controlling a robot on the ground, which requires lower dynamic stability from the algorithm compared with jumping actions. However, for multi-joint robots, the high dimensionality of the action space makes it difficult for the reward function to converge. Moreover, extensive exploration during the learning process makes it difficult for the robot to learn specific skills. 

In this study, we focused on the stable jumping task in a highly dynamic environment, aiming to develop an intelligent control algorithm for biologically inspired robots. The main contributions of this study are specifically summarized as follows:

- We present a stable jumping control algorithm (JCA) based on deep reinforcement learning. The algorithm employs an actor–critic architecture to learn a control policy, mapping the robot’s observations (robot position and velocity, obstacle position, target position, etc.) to its joint torques. We introduce dual Q-network and entropy regularization to increase exploration and randomness in the learning policy.
- Inspired by the locomotion skills of insects, we developed a stage incentive mechanism to adjust the reward function dynamically, which greatly improves the robot’s jumping stability and accuracy.
- We implemented the Stephenson six-bar mechanism within a simulation environment to establish a locust-inspired jumping robot platform.
- Extensive simulations were conducted, demonstrating the effectiveness of the proposed methods. The jumping robot could perform smooth, non-flip jumps, with the error of the distance from the target point remaining below 3%, and it consumed 44.6% less energy to travel the same distance by jumping compared with walking. Furthermore, the proposed algorithm exhibited a faster convergence rate and improved convergence effects compared with other classical algorithms.

The remainder of this paper is organized as follows: In Section 2, the structure and design of the jumping robot is presented. In Section 3, we present an explanation of the problem formulation. In Section 4, we provide a detailed description of the stable jumping control algorithm. In Section 5, we present the simulation results, followed by a comprehensive discussion. In Section 6, we summarize the conclusions of our work and discuss future research directions.

## 2. Design of the Jumping Robot

**Figure 1:**
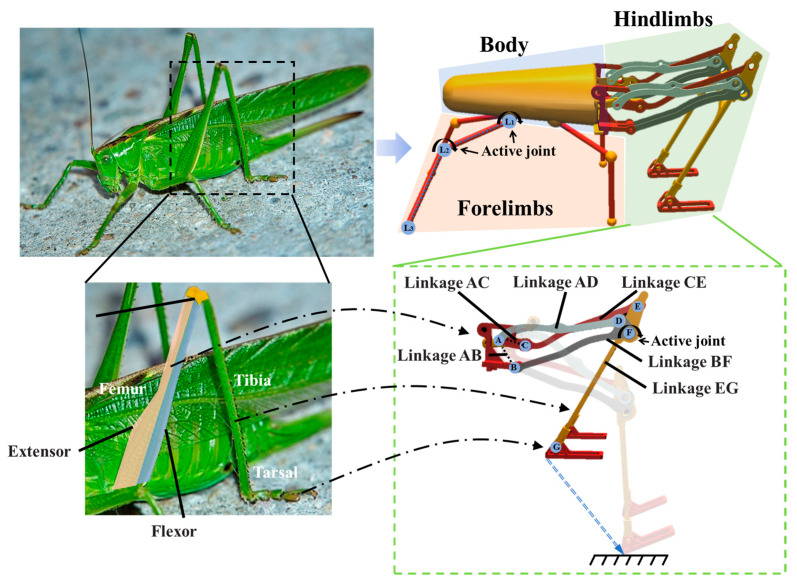
Configuration and design of the jumping robot. Each hindlimb of the jumping robot contains six passive joints at A, B, C, D, E, G and one active joint at F. (The locust image is sourced from a free and open-source media website, “https://pixabay.com/zh/photos/grasshopper-court-garden-green-5394624 (accessed on 25 August 2024)”).

Many insects in the order Orthoptera, such as locusts, crickets, and grasshoppers, exhibit remarkable jumping abilities because of their similar leg structures. In our previous work [6,26], we conducted an analysis of a locust skeletal muscle model and developed a biomimetic jumping leg mechanism based on the Stephenson six-bar mechanism, as shown in Figure 1. This mechanism recalls the leg structure of locusts, with extensor and flexor muscles corresponding to linkages AD and CE, respectively, and the femoral, tibial, and tarsal segments corresponding to linkage BF, linkage ED, and the additional linkage provided at passive joint G, respectively. Unlike locusts, the robot’s two hindlimbs are connected by linkage ED. We optimized the Stephenson six-bar mechanism to ensure that the trajectory of the foot end is approximately linear, thereby providing favorable conditions for stable jumping. In Figure 1, we show the trajectory of the end movement of the leg mechanism from contraction to extension. 

Based on the leg mechanism, we designed a biologically inspired jumping robot comprising the following three main components: the forelimbs, the body, and the hindlimbs. The jumping robot has four forelimbs and two hindlimbs. Each of the four forelimbs consists of two linkages in series, driven by two active joints (L1 and L2). Each hindlimb, based on the Stephenson six-bar mechanism, contains six linkages and six passive joints. The two hindlimbs are controlled by one active joint at F, which accomplishes the contraction and extension process of the hindlimbs. In simulation, the actuator of the active joint is modeled as an ideal motor capable of delivering the desired torque in any time step. The dimensions of the jumping robot are 17.7 cm in length, 7.9 cm in width, and 8.2 cm in height.

## 3. Problem Formulation

By emulating the leg structure of locusts, we aimed to leverage the natural mechanisms that contribute to their jumping performance. In comparison with the serial leg mechanisms commonly found in humanoid robots and other legged robots, this leg mechanism incorporates a greater number of passive joints, which introduces complexity in both modeling and control, i.e., it increases the degrees of freedom, which makes achieving precise control over the robot’s motion more challenging. In this study, we mapped this complicated leg mechanism into a simulation environment and established the robot platform to achieve stable jumping based on deep reinforcement learning.

To emphasize the characteristics of the jumping robot, its actions are classified into three types as follows: jumping over obstacles, jumping down from obstacles, and jumping up on obstacles. In each time step, the jumping robot decides the joints’ torque from the observed information. The goal of the mission is to enable the jumping robot to jump smoothly over to the target position. The motion decision process can be defined as a Markov decision process (MDP), which is represented as a five-tuple $\left(S,A,P,R,\gamma \right)$, where S represents the state space, A represents the action space, and R:S×A→r is the reward function. Moreover, $P:S\times A\times S\to \left[0,1\right]$ represents the state transition probability distribution and $\gamma \in \left[0,1\right]$ denotes the discount factor. Given an MDP, DRL aims to find a policy π:S→A to maximize the expected future discounted reward $E_{\pi }\left[\sum_{t=0}^{\infty }\gamma^{t}R_{t}\right]$, where $E_{\pi }\left[\cdot \right]$is the expectation operator. 

## 4. Stable Jumping Control Algorithm

In this section, the proposed jumping control algorithm is introduced, with details given for each of its three main parts including the observation and action space, the stage incentive mechanism, and the algorithmic framework. First, we describe the design of the simulation environment, focusing on the following two main components: the observation space and the action space. The stage incentive mechanism was designed to ensure that the robot can perform various types of stable jumps. The algorithmic framework was developed to enhance convergence effects and improve the convergence rate.

### 4.1. Action and Observation

Based on the configuration and design of the jumping robot, the robot has nine active joints, each of which is equipped with a torque actuator. The cooperation of all actuators allows for stable jumping motion. Therefore, the jumping robot employs nine torque actuators in each time step in the interaction environment. The continuous action space of the robot consists of a one-dimensional array $A=\left\{\tau_{1},\tau_{2},\ldots ,\tau_{9}\right\}$. In addition, we normalize and scale the torque range for each actuator to [−1, 1].

The accurate design of the state space is essential for network training in deep reinforcement learning. Based on the defined task, the observation space contains information about the various states of the jumping robot, the target position, and the obstacle distance for action decision-making. It is divided into five main parts as follows:

- The position and posture of the robot;
- The velocity of the robot in the x-, y-, and z-axis directions;
- The rotational angles and angular velocities of the robot’s joints;
- The target position;
- The obstacle position.

### 4.2. Stage Incentive Mechanism

The reward function determines whether the stable jumping control of the robot can be accomplished effectively. The jumping action is the most difficult action to learn because the robot not only has to approach the target position but also has to maintain a stable aerial posture. Therefore, it is essential to design a reasonable reward mechanism to guide the learning process of the jumping robot. Insects exhibit a high degree of synergy between the movements of their forelimbs and hindlimbs during various stages of the jump process. Inspired by the biological motion skills of insects, we divided the robot’s jump process into three stages as follows: take-off, soaring, and landing. In each stage, we established corresponding incentive mechanisms to adjust reward functions dynamically, which can be decomposed into four parts.

The first part, used throughout the jump process, aims to encourage the jumping robot to approach the target position. The smaller the distance between the robot and the target, the greater the reward obtained. Therefore, the reward for the robot that tracks the target is as follows:

(1) $$R_{tar}=-dist\left(d_{i},d_{T}\right)$$

where $dist\left(d_{i},d_{T}\right)$ denotes the error of the Euclidean distance between the jumping robot and the target position.

Jumping is one of the most common movements of locusts. However, studies have shown that these insects are susceptible to rotation during jumping because of the rapid change in their posture [27]. Rotation is produced by positive and negative torque acting on the insect during flight and after take-off. Specifically, negative torque occurs when the center of mass (COM) lies below the plane of the thrust vector during take-off, causing head-down rotation. Certain insects employ distinctive biological motion skills to address the challenge of rapid attitude change when jumping. The flexion of the longitudinal muscles of the forelimb or hindlimb action creates positive torque, which counteracts the head-down rotation. Spiders [28] and praying mantises [29] control their rotation during take-off by counter-rotating their forelimbs and hindlimbs to maintain balance. The second part of the reward function is inspired by these cases and aims to achieve the cooperation of the forelimb and hindlimb joints when the robot performs jumping actions as follows:

(2) $$R_{coo}=\left\{\begin{array}{cc} V_{f}+V_{h}, & n\leq 5\\ 0, & n\geq 5 \end{array}\right.,$$

where $V_{f}$ and $V_{h}$ denote the rotational velocities of the forelimb and hindlimb joints, respectively, and n is the time step. This component of the reward function influences the robot’s take-off stage, aiming to ensure that the robot’s center of mass aligns with the thrust vector.

It is essential to prevent the robot from flipping. The most effective approach to achieve this is to minimize the pitch, roll, and yaw angular velocities. If these parameters have significant values, the jumping robot incurs a substantial penalty. The third part of the reward function thus ensures that the robot’s posture maintains its stability during a jump as follows:

(3) $$R_{sta}=-|V_{pitch}|-|V_{roll}|-|V_{yaw}|,$$

where $V_{pitch}$, $V_{roll}$, and $V_{yaw}$ denote the pitch, roll, and yaw angular velocities of the robot, respectively.

Locusts take time to restore energy after each jump; however, in a simulation environment, the robot does not need to do so. For the robot to be able to perform the next jump quickly and land smoothly, we want it to complete the contraction of its hindlimbs during the jump and return to the starting stance. Thus, when the robot reaches a certain height, it is penalized if its hind limbs do not contract:

(4) $$R_{rec}=-\theta_{h},H>h,$$

where $\theta_{h}$ denotes the angle of the robot’s hindlimb joints. H is the current height of the robot, and h is a constant for the height setting. This part of the reward function is implemented in both the soaring and landing stages.

Hence, the comprehensive reward of SMC at each step is expressed as follows:

(5) $$R=\left[\begin{array}{cccc} \eta_{1} & \eta_{2} & \eta_{3} & \eta_{4} \end{array}\right]\cdot {\left[\begin{array}{cccc} R_{tar} & R_{coo} & R_{sta} & R_{cov} \end{array}\right]}^{T},$$

where $\eta_{1},\eta_{2},\eta_{3},\eta_{4}$ are the coefficients that balance the weights of each part of the reward function.

### 4.3. The Framework of the Jumping Control Algorithm

**Figure 2:**
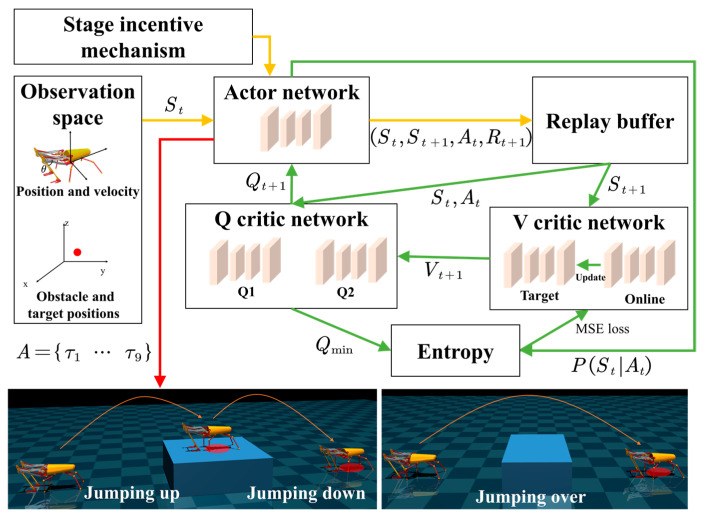
The framework of the jumping control algorithm. The yellow arrows indicate the acquisition process of the experience generated by the robot’s interaction with the environment. The green arrows indicate the training process of the entire network. The red arrow indicates the execution process of the trained policy network.

The actor–critic architecture combines value-based and policy-based DRL algorithms for more efficient learning of the appropriate policy in various complex environments. However, when the observation space or action space is too large, it is difficult for each action’s value to be estimated and for the training process to converge. The Soft Actor–Critic (SAC) [30] algorithm trains the policy to increase randomness and exploration by introducing entropy regularization, which has been shown to yield excellent performance in robot control tasks. Therefore, we developed a jumping control algorithm based on SAC, as shown in Figure 2. By modeling the jumping robot and its jumping environment, the robot can collect data from the observation space. The actor network employs the current policy to determine the actions to be performed. By utilizing a stage incentive mechanism to adjust reward functions dynamically, the robot learns animal behavior and receives rewards after performing actions. A replay buffer is then utilized to store the experiences generated by the robot’s interaction with the environment, including states, actions, rewards, and the next states. These experiences are randomly sampled from the replay buffer, which helps to disrupt the temporal correlation among them and increase learning efficiency. The V-critical network is used to estimate the value of a state and consists of an online network and a target network, where the latter is a slightly delayed version of the former and is periodically updated to stabilize the learning process. The Q critic network, consisting of two identically structured networks, is used to estimate the value of performing an action in a state. Additionally, entropy regularization is applied to increase randomness and exploration within the network. This approach introduces an entropy term to the optimal policy, which penalizes certainty in the action distribution. Both the actor and critic networks are constructed by using the Multi-Layer Perceptron (MLP) architecture, which incorporates fully connected layers and activation functions. Upon network training completion, the actor network functions as a policy network, outputting joint torques in each time step to enable the robot to perform jumping tasks.

Unlike standard DRL, SAC trains a stochastic policy with entropy regularization that maximizes not only the expected return of the policy but also the entropy of the policy. Hence, the optimal policy is given by:

(6) $$\pi^{*}=\arg\underset{\pi }{\max}\sum_{t=0}^{\infty }E_{\pi }\left[\gamma^{t}R_{t}\left(S_{t},A_{t}\right)+\alpha H\left(\cdot |S_{t}\right)\right],$$

where $S_{t}$ is the state observation and $A_{t}$ is the action selected for each interaction *t*. In addition, $H\left(\cdot |S_{t}\right)$ is the entropy of the policy, and α controls the balance between exploration and exploitation. A stochastic policy has greater exploration capabilities than a deterministic policy. The range of solutions for a continuous action space is very large, but because of the rapid change in robot attitude during jumping, extreme precision in action selection is required to ensure posture stabilization. Therefore, it is particularly useful for highly dynamic motion control in robots. 

As in standard DRL, the state value function $V_{\pi }\left(S_{t}\right)$ denotes the expected return of policy π in state $S_{t}$. Q-function $Q_{\pi }\left(S_{t},A_{t}\right)$ denotes the expected return of policy π under the conditions of state $S_{t}$ and action $A_{t}$. We parameterize the value function $V_{\vartheta }\left(S_{t}\right)$ by using neural networks with parameters ϑ. To prevent the overestimation of the Q-value, the Q-function is parameterized by using two neural networks $Q_{\psi_{1}}\left(S_{t},A_{t}\right)$ and $Q_{\psi_{2}}\left(S_{t},A_{t}\right)$ with parameters $\psi_{1}$ and $\psi_{2}$. The value function networks are trained to minimize the squared residue error as follows:

(7) $$J_{V}\left(\vartheta \right)=E_{S_{t}\sim D}{\left[V_{\vartheta }\left(S_{t}\right)-V_{soft}\left(S_{t}\right)\right]}^{2},$$

(8) $$V_{soft}\left(S_{t}\right)=E_{\pi }\left[\min\left(Q_{\psi 1}\left(S_{t},A_{t}\right),Q_{\psi 2}\left(S_{t},A_{t}\right)\right)\right]+E_{\pi }\left[\alpha \log_{\pi }\left(A_{t}|S_{t}\right)\right].$$

In addition, the Q-function networks are trained to minimize the squared residue error as follows:

(9) $$J_{Q}\left(\psi \right)=\left\{\begin{array}{l} E_{S_{t},A_{t}\sim D}{\left[Q_{\psi 1}\left(S_{t},A_{t}\right)-Q_{soft}\left(S_{t},A_{t}\right)\right]}^{2},\psi =\psi_{1}\\ E_{S_{t},A_{t}\sim D}{\left[Q_{\psi 2}\left(S_{t},A_{t}\right)-Q_{soft}\left(S_{t},A_{t}\right)\right]}^{2},\psi =\psi_{2} \end{array}\right.,$$

(10) $$Q_{soft}\left(S_{t},A_{t}\right)=R\left(S_{t},A_{t}\right)+\gamma E_{S_{t+1}}\left[V_{\bar{\vartheta }}\left(S_{t+1}\right)\right],$$

where D denotes the distribution of previously sampled $S_{t}$ and $A_{t}$, and $V_{\bar{\vartheta }}$ denotes the target value network, which updates the target weights to match the current value network weights periodically. Finally, the actor network can be trained as shown in (9).

(11) $$J_{\pi }\left(\phi \right)=\frac{1}{\left|B\right|}E\left(Q_{\varphi_{1}\;or\;\varphi_{2}\;}\left(S_{t},\tilde{A}\right)-\alpha \log\left(\pi_{\phi }\left(\tilde{A}|S_{t}\right)\right)\right),$$

where $\tilde{A}\sim \pi \left(\cdot |S_{t}\right)$denotes all possible actions predicted by the actor network based on policy π. Moreover, B represents a randomly minibatch sample; taking the average value can reflect the quality of the samples taken in the average sense. The overall training process for the jumping control algorithm is presented in Algorithm 1.
**Algorithm 1.** Jumping control algorithm **Initialize:** Actor network parameters ϕ, Q-network parameters $\psi_{1}$ and $\psi_{2}$, online V network parameters ϑ, target V critic network parameters $\bar{\vartheta }$, experience replay buffer *D*.
**for** episodes = 1 **to** $episode_{max}$ **do**Reset jumping robot and receive initial states**for** *t* = 1 **to** start-timesteps **do**Execute action $A_{t}=\left\{\tau_{1},\tau_{2},\ldots ,\tau_{9},\right\}$ to receive reward $R=\left[\begin{array}{cccc} \eta_{1} & \eta_{2} & \eta_{3} & \eta_{4} \end{array}\right]\cdot {\left[\begin{array}{cccc} R_{1} & R_{2} & R_{3} & R_{4} \end{array}\right]}^{T}$ and next state $S_{t+1}$Store $\left(S_{\begin{array}{c} t \end{array}},S_{\begin{array}{c} t \end{array}+1},A_{t},R_{t+1}\right)$ in the experience replay buffer *D***end for****for each gradient step do**Randomly sample a minibatch B from *D*Update ϑ by minimizing $J_{V}\left(\vartheta \right)=E_{S_{t}\sim D}{\left[V_{\vartheta }\left(S_{t}\right)-V_{soft}\left(S_{t}\right)\right]}^{2}$Update $\psi_{1}$ by minimizing $E_{S_{t},A_{t}\sim D}{\left[Q_{\psi_{1}}\left(S_{t},A_{t}\right)-Q_{soft}\left(S_{t},A_{t}\right)\right]}^{2}$Update $\psi_{2}$ by minimizing $E_{S_{t},A_{t}\sim D}{\left[Q_{\psi_{2}}\left(S_{t},A_{t}\right)-Q_{soft}\left(S_{t},A_{t}\right)\right]}^{2}$Update ϕ by minimizing $\frac{1}{\left|B\right|}E\left(Q_{\psi_{1}\;or\;\psi_{2}\;}\left(S_{t},\tilde{A}\right)-\alpha \log\left(\pi_{\phi }\left(\tilde{A}|S_{t}\right)\right)\right)$**if** update target critic network, **then** $\bar{\vartheta }\leftarrow \tau \vartheta +\left(1-\tau \right)\bar{\vartheta }$**end for****end for**


## 5. Simulation and Analysis

In this section, we introduce the simulation environment constructed and the extensive simulations performed to validate the effectiveness of the proposed algorithm. First, we performed jumping experiments with different distances based on the proposed algorithm. Then, we conducted experiments comparing the proposed algorithm with other classical algorithms. Finally, we performed experiments comparing jumping and walking actions. All simulations were performed on a laptop equipped with an AMD Ryzen 9-7845HX CPU (Advanced Micro Devices, Inc., Santa Clara, California, United States) and an Nvidia RTX 4070 GPU (Nvidia Corporation, Santa Clara, California, United States). 

### 5.1. Jumping Experiments with Different Types

We built our locust-inspired jumping robot model in the free and open-source physics engine MuJoCo [31]. The jumping types were divided into jumping over obstacles, jumping down from obstacles, and jumping up on obstacles. For each type, the robot, which kept the same initial state, was trained on different jump distances of 40 cm, 50 cm, 60 cm, and 70 cm. In the jumping-over experiments, the distance between the obstacle and the center of the robot was set to 20 cm.; additionally, the width of the obstacle was set to 6 cm to ensure adequate jumping space. In the jumping-up experiments, the distance between the obstacle and the center of the robot was set to 30 cm. In the jumping-down experiments, the robot was on top of the obstacle, and the distance from the obstacle was 0. Videos of jumping experiments with different types are shown in the Supplementary Materials, Video S1. The key parameters of the proposed algorithm can be seen in Table 1.

**Figure 3:**
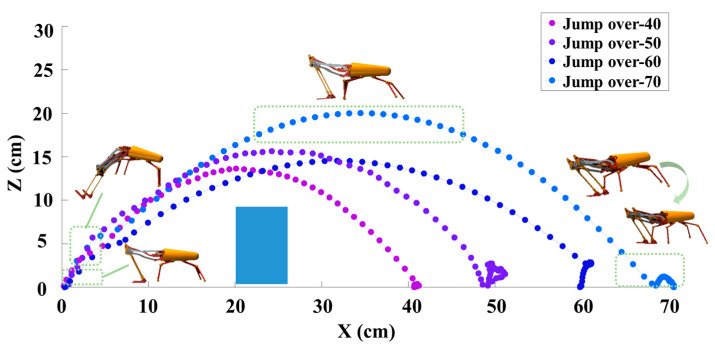
Trajectories of the robot jumping over obstacles at different distances.

We conducted experiments by using our proposed algorithm to evaluate the robot’s ability to jump over obstacles at the distances of 40 cm, 50 cm, 60 cm, and 70 cm; the results are shown in Figure 3. As noted earlier, the whole jump process can be divided into three stages, which are take-off, soaring, and landing. The jump trajectories show that the robot could complete the whole jump process smoothly over different jump distances. While fluctuations occurred upon landing, the robot consistently reached the target distance with each jump. It is important to note that the jumping robot did not collide with the obstacle, even when they were in close proximity to one another. 

**Figure 4:**
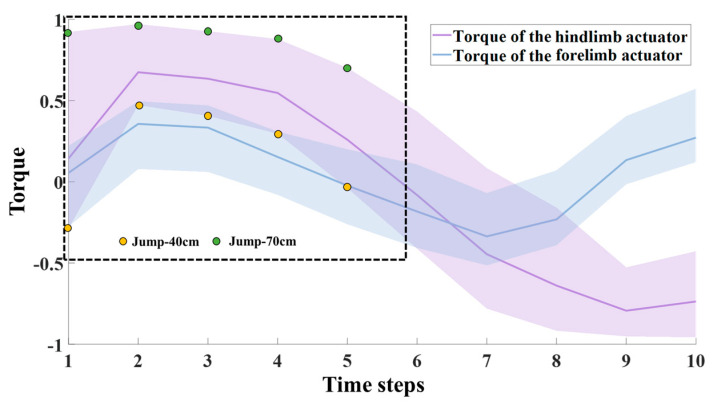
Torque output curves of forelimb and hindlimb actuators at different distances during the take-off stage.

During the take-off stage, the robot’s hindlimb and forelimb actuators rotated rapidly to push the robot off the ground. To demonstrate the collaboration between the forelimbs and hindlimbs, torque output curves of their actuators at different distances are shown in Figure 4. The curves indicate that the torque outputs of the forelimb and hindlimb actuators exhibited similar trends. This is due to the incorporation of the stage incentive mechanism in our algorithm, which enables the robot to learn biological motion skills. The ability of the robot’s forelimbs and hindlimbs to work together ensures that the robot’s action is smooth during the take-off stage. Additionally, the figure demonstrates that the torque output of the robot’s hindlimb actuators for a 40 cm jump is lower than that for a 70 cm jump.

**Figure 5:**
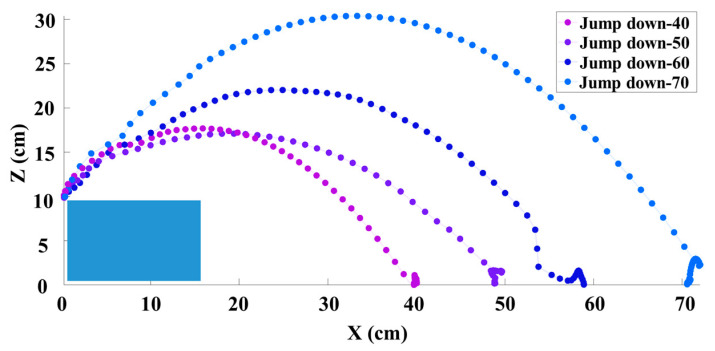
Trajectories of the robot jumping down from obstacles at different distances.

**Figure 6:**
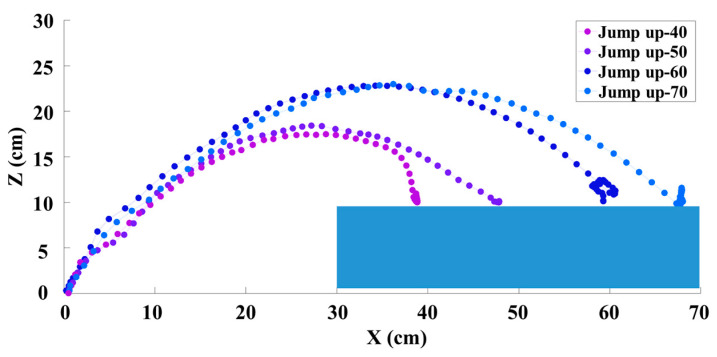
Trajectories of the robot jumping up on obstacles at different distances.

Then, we conducted experiments by using our algorithm to evaluate the robot’s ability to jump down from obstacles and jump up on obstacles at distances of 40 cm, 50 cm, 60 cm, and 70 cm; the results are shown in Figure 5 and Figure 6, respectively. As we can see, some of the trajectories of the jumps over different distances are not perfect parabolas. This is because, during the jump process, the robot aimed to reach the target position and autonomously adjusted its center of mass during the soaring stage through joint movements, resulting in changes in the trajectory.

**Figure 7:**
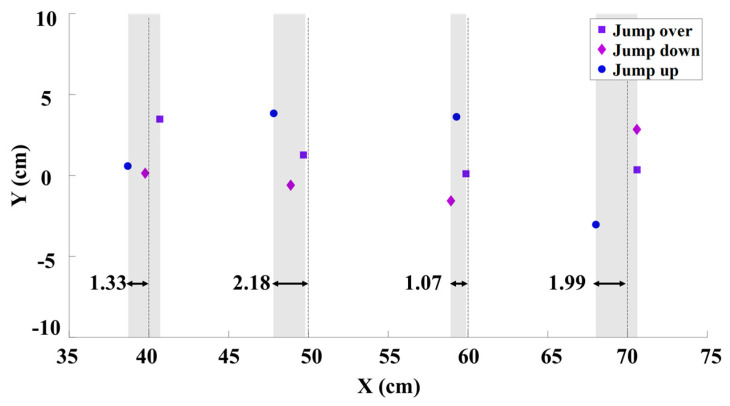
Landing errors at different jump distances.

In Figure 7, the landing errors of the jumping robot for the three jump types and four jump distances are illustrated. Although the robot could land close to the target position each time, some distance errors occurred. This is because, to increase the robustness of the algorithm, we introduced random noise into the robot’s initial state for each jump. The error–distance ratios are less than 3%, which is acceptable for the deployment task. Specifically, the maximum distance error is 2.18 cm, and the ratio of the robot’s jump distance to the distance error is below 3%, indicating an excellent result.

**Figure 8:**
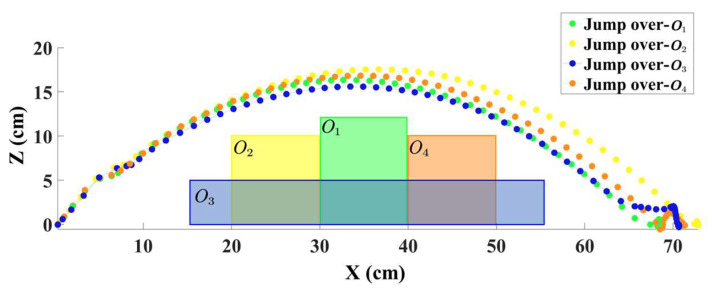
Trajectories of the robot jumping over different obstacles.

To demonstrate the stability and effectiveness of the algorithm, we conducted experiments involving the robot jumping over different obstacles; the trajectories are shown in Figure 8. The width and height of obstacles $O_{1}$, $O_{2}$, $O_{3}$, and $O_{4}$ were set to 10 cm and 12 cm, 10 cm and 10 cm, 40 cm and 5 cm, and 10 cm and 10 cm, respectively. The distances from the robot’s center to the center of obstacles $O_{1}$, $O_{2}$, $O_{3}$, and $O_{4}$ were set to 35 cm, 25 cm, 35 cm, and 45 cm, respectively. As shown in the figure, despite the presence of various types of obstacles, the robot was capable of learning an effective control policy to complete the jumping task successfully.

### 5.2. Jumping Experiments with Different Algorithms

**Figure 9:**
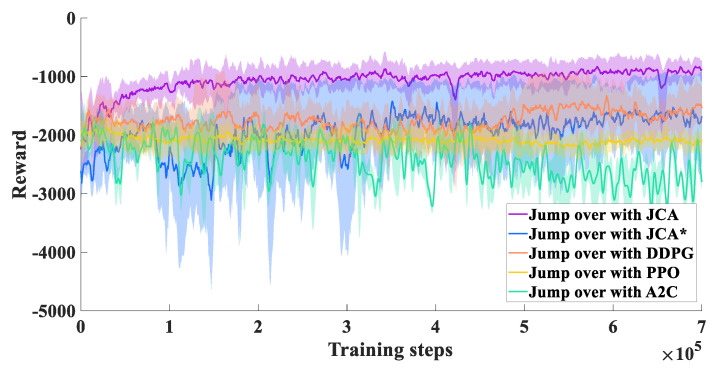
Learning curves of models with JCA, JCA* (without SIM), DDPG, PPO, and A2C.

**Figure 10:**
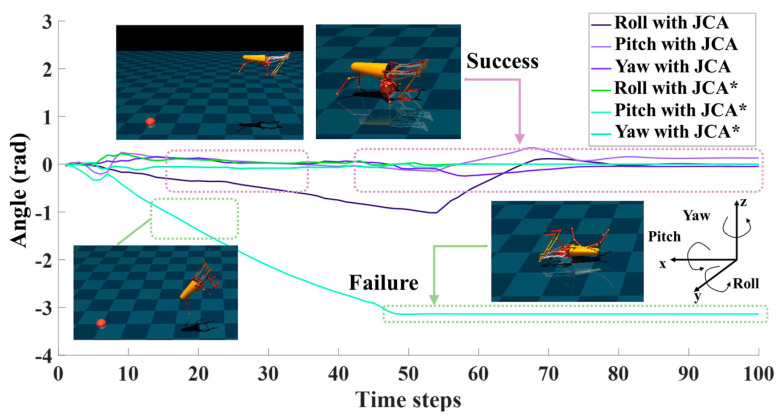
Experimental results for JCA and JCA* (without SIM). The purple arrow indicates that JCA enables the robot to perform non-flip jump. The green arrow indicates that JCA* is unable to make the robot perform a non-flip jump.

To show the advantages of the proposed algorithm, we set the same environmental parameters and performed a comparison with the PPO and DDPG algorithms. The experimental results show that the JCA improved stability during the training process by using dual Q-network and target entropy regularization. Compared with DDPG, PPO, and A2C, the JCA had a faster convergence rate and improved convergence effects, as shown in Figure 9. All the above jumping experiments were conducted under the guidance of a stage incentive mechanism (SIM). For comparison, we also performed jumping experiments without a SIM, where the algorithm is denoted as JCA*. With the JCA*, convergence was clearly lower compared with the JCA, as shown in Figure 9. Without a SIM, the jumping robot could reach the vicinity of the target point, but it tended to flip in the air and was not able to reach the target smoothly, while with a SIM, although there were variations in the roll angle of the robot after takeoff, the jumping robot could adjust its body to maintain a stable state within controllable limits before landing, as shown in Figure 10. Videos of jumping experiments with different algorithms are shown in the Supplementary Materials, Video S1.

### 5.3. Comparative Experiments of Jumping and Walking

**Figure 11:**
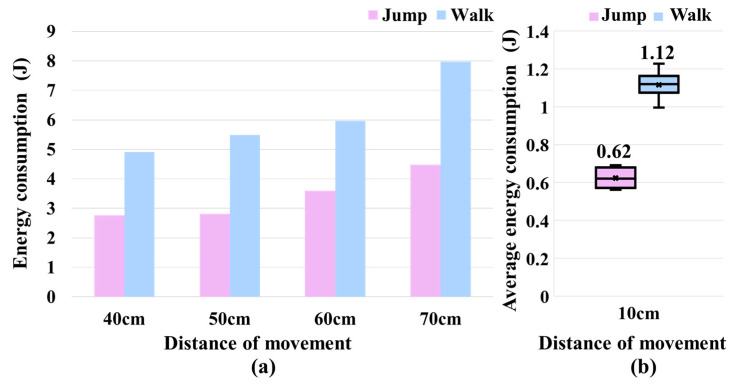
Comparison of energy consumption during jumping and walking actions. (**a**) Comparison of energy consumption at different movement distances. (**b**) Comparison of average energy consumption per 10 cm for jumping and walking.

We also used the proposed algorithm to make the robot perform a walking action. We recorded the output torque and angular velocity of all actuators when completing the entire movement and calculated the energy consumption. We then compared the experimental results of jumping over obstacles with those of walking. Given that the starting and ending points for both the robot’s walking and obstacle-jumping experiments lay on the same plane, such configuration was deemed appropriate. As Figure 11a shows, when the robot traveled the same distance, the energy consumption of the jumping action was much lower than that of the walking action. In addition, for jumping and walking, we calculated the average energy consumption for a movement of 10 cm, as shown in Figure 11b. On average, the energy consumption for jumping was about 44.6% lower than that for walking.

### 5.4. Discussion

In summary, we propose a stable jumping control algorithm for a locust-inspired jumping robot based on deep reinforcement learning. This algorithm enables the robot to achieve smooth, non-flip jumps successfully. This may be attributed to the following reasons. First, the SIM module increases the stability of the robot when jumping. Studies have indicated that in insects, during the takeoff phase, if the center of mass is situated below or above the plane of the thrust vector, an insect is prone to imbalance during the soaring phase, resulting in flipping upon landing [27,28,29]. Therefore, we introduced SIM guidance in the training process to ensure that the center of mass lies within the plane of the thrust vector, thus reducing the flipping torque. Our experiments indicate that this addition increases the stability of the jump process by coordinating the output of torque from the forelimbs and hindlimbs to alter the thrust direction. The jumping robot can, therefore, maintain balance when jumping over, down, or up on obstacles. 

In future work, we will develop a physical prototype of the designed jumping robot that includes a perception module and a control module. Additionally, physical experiments will be conducted to further validate the effectiveness of the proposed method. Based on the data obtained from the simulation, we predict that the physical prototype robot will need to achieve a power-to-weight ratio of approximately 0.4 W/g. According to the literature, this ratio ranges from 0.2 to 0.5 W/g in locusts [4,32,33]. Thus, the parameters of the robot would closely resemble those of locusts. To achieve this goal, we also need to design more compact and efficient energy storage structures for the jumping robot.

## 6. Conclusions

In this work, we developed a jumping control algorithm to improve the stability and accuracy of a locust-inspired jumping robot. Based on deep reinforcement learning, we constructed an actor–critic network architecture to achieve motion control by mapping the robot’s observations to its joint torques. In order to improve the stability and efficiency of the algorithm, we introduced double Q-network and target entropy regularization. In addition, inspired by the biological motion skills of insects, a stage incentive mechanism was designed based on three stages of robotic jumping to maintain dynamic stability. Then, we built a simulation environment and created a locust-inspired jumping robot platform. Finally, extensive simulations were conducted, demonstrating the effectiveness and performance of the proposed algorithm.

## Figures and Tables

**Table 1:** The parameters of jumping control algorithm.

Item	$\eta_{1}$	$\eta_{2}$	$\eta_{3}$	$\eta_{4}$	h
Value	2.5	10	5	5	15 cm

## Data Availability

The data are contained within the article.

## Supplementary Materials

The following supporting information can be downloaded at: https://www.mdpi.com/article/10.3390/biomimetics9090548/s1, Video S1: A Locust-inspired jumping robot for different types of jumping experiments.
